# Connection of pre-competition anxiety with gut microbiota and metabolites in wrestlers with varying sports performances based on brain-gut axis theory

**DOI:** 10.1186/s12866-024-03279-4

**Published:** 2024-04-27

**Authors:** Pengyu Fu, Cuiping Wang, Shuai Zheng, Lei Qiao, Weiyang Gao, Lijing Gong

**Affiliations:** 1 Department of Physical Education, Northwestern Polytechnical University, Xi’an, 710072 Shaanxi China; 2 College of Sports and Health Sciences, Xi’an Physical Education University, Xi’an, 710068 Shaanxi China; 3 College of Life Science, Northwestern Polytechnical University, Xi’an, 710072 Shaanxi China; 4 School of Languages and Cultural Communication, English Department, Xi’An Mingde Institute of Technology, Xi’an, 710124 Shaanxi China; 5 Key Laboratory of Exercise and Physical Fitness, Ministry of Education, Beijing Sport University, Beijing, 100084 China

**Keywords:** Wrestlers, Pre-competition anxiety, Brain-gut axis, Gut microbiota, Untargeted metabonomics

## Abstract

**Objective:**

The purpose of this study is to investigate the connection of pre-competition anxiety with gut microbiota and metabolites in wrestlers with different sports performances.

**Methods:**

One week prior to a national competition, 12 wrestlers completed anxiety questionnaires. Faecal and urine samples were collected for the analysis of gut microbiota and metabolites through the high-throughput sequencing of the 16 S rRNA gene in conjunction with untargeted metabolomics technology. The subjects were divided into two groups, namely, achievement (CP) and no-achievement (CnP) wrestlers, on the basis of whether or not their performances placed them in the top 16 at the competition. The relationship amongst the variations in gut microbiota, metabolites, and anxiety indicators was analyzed.

**Results:**

(1) The CP group exhibited significantly higher levels of “state self-confidence,” “self-confidence,” and “somatic state anxiety” than the CnP group. Conversely, the CP group displayed lower levels of “individual failure anxiety” and “sports competition anxiety” than the CnP group. (2) The gut microbiota in the CP group was more diverse and abundant than that in the CnP group. Pre-competition anxiety was linked to *Oscillospiraceae UCG_005*, *Paraprevotella*, *Ruminococcaceae* and *TM7x*. (3) The functions of differential metabolites in faeces and urine of the CP/CnP group were mainly enriched in caffeine metabolism, lipopolysaccharide biosynthesis and VEGF and mTOR signaling pathways. Common differential metabolites in feces and urine were significantly associated with multiple anxiety indicators.

**Conclusions:**

Wrestlers with different sports performance have different pre-competition anxiety states, gut microbiota distribution and abundance and differential metabolites in faeces and urine. A certain correlation exists between these psychological and physiological indicators.

## Introduction

Pre-competition anxiety, which is the primary factor influencing sports performance, is the psychological and bodily distress induced by uncertainty about a competition’s outcome and other variables prior to the competition. It is a state that requires evaluation by using multidimensional indicators, including self-confidence, state anxiety, trait anxiety, physical anxiety and cognitive anxiety. The levels of these indicators have different relationships with sports performance. Generally speaking, inverted U curve theory [1] demonstrates that performance can be negatively affected by anxiety levels that are either excessively high or excessively low and that maintaining an optimal degree of nervousness in essential to perform well [2, 3].

In addition to regulating metabolism, endocrine and immune functions, the trillions of microorganisms in the intestines have also been proven to affect human mood and behaviour by regulating neurotransmitters, thus linking the intestines with the central nervous system. This link is a key part of brain–gut axis theory [4]. Physical and mental stress induced by exercise has been reported to be strongly correlated with changes in gut microbiota composition [5, 6]. Although the relationship between pre-competition anxiety and the gut microbiome has not been thoroughly studied, an increasing number of studies on humans and animals have shown that anxiety could affect the abundance, composition, and metabolite levels of certain gut microbes. Additionally, works have found that adding probiotics reduces anxiety and enhances performance [7–10]. However, none of these studies involved wrestling, especially the relationship between anxiety and the gut microbiome before a certain competition.

Modern wrestling mainly relies on the anaerobic energy system, which employes numerous efforts and is accompanied by the accumulation of lactic acid. Exercise fatigue develop as a result of increasing training frequency and intensity before to competition [11]. To obtain an advantage in lower weight category, wrestlers commonly engage in rapid weight loss, which has been demonstrated that negatively impacts wrestlers’ mood and health [12]. The above factors lead to pre- competition anxiety in wrestlers. This anxiety triggers physiological and psychological stress reactions, and then activates the hypothalamus-pituitary-adrenal (HPA) and sympatho-adrenomedullaty (SAM) axis, which secretes catabolic and stress hormones as well as inflammatory cytokines that affect the digestive system [13], including gut microbiota and metabolites. We hypothesized that these psychological and physiological changes affect a wrestler’s sport performance. However, reports on this topic have not yet been found.

Given the complexity of assessing pre-competition anxiety states, using the presence of anxiety as a grouping criterion is challenging. Therefore, in this study, wrestlers were divided into two groups on the basis of competition performance and differences in pre-competition anxiety levels and gut microbiota. Their metabolites and correlations were analysed to determine the effect of these two factors on the performance of wrestlers. The goal of this study is to broaden the meaning of brain–gut axis theory in competitive sports and provide a theoretical foundation for enhancing the performance of wrestlers. Investigating the connection between the gut microbiota and anxiety in wrestlers prior to competition, may aid in developing a sensible food plan and offer advice on selecting probiotics and sports nutrition supplements.

## Materials and methods

### Study objects and groups

This study involved 12 high-level wrestlers, who were training at the Shaanxi Provincial Sports Training Center, at the period of preparation for the Chinese International Wrestling Championships in 2022, involved in this study. The wrestlers aged 20 ± 1.76 years old, had been accepted the professional training for 6.67 ± 1.76 years. The weight category was 68.17 ± 5.34 kg for male wrestlers and 63.83 ± 7.65 kg for female wrestlers. The wrestlers shared a diet, lifestyle (lived and ate together) and pre-competition training regimen and aimed to lose some weight before the competition. Subjects were excluded if they (1) were unable to train because of a recent injury; (2) used antibiotics and probiotic supplements in the previous 6 months; and (3) had gastrointestinal diseases, such as diarrhea and constipation. They were divided into two groups on the basis of whether they placed in the top 16: (1) achievement group (CP group, *n* = 6) and (2) no-achievement group (CnP group, *n* = 6). The wrestlers in the CP group ranked 3rd, 9th, 11th and 15th in the female division and 11th and 16th in the male division. The CnP group included four male and two female wrestlers, respectively. The Medical and Experimental Animal Ethics Committee of Northwestern Polytechnical University approved this study (approval number: 202,202,042), and all wrestlers provided signed informed consent forms prior to the experiment.

### Questionnaire for pre-competition anxiety

One week before the competition, the wrestlers completed paper versions of the four following questionnaires:

1) The Competition State Anxiety Inventory-2 (CSAI-2) [14] is divided into three subscales: cognitive state anxiety, somatic state anxiety and state self-confidence. The questionnaire includes 27 questions, such as ‘I am worried about this competition’, with scores ranging from 1 to 4 points (from weak to strong) depending on the current intensity of the feeling.

2) The Pre-competition Emotion Scale-T (PES-T-32 × 6) [15] is divided into four subscales: individual failure anxiety, self-confidence, social expectation anxiety and somatic anxiety. This questionnaire contains 32 questions, such as ‘I am worried that I will not be able to compete as well as usual’, with scores ranging from 1 to 6 points (from less to more) depending on the frequency of the feeling before competition.

3) The Sports Competition Anxiety Scale (SCAT) [15]. The questionnaire contains 15 questions, such as ‘I am happy when I compete with my competitors’, with scores ranging from 1 to 3 points (from less to more) depending on the frequency of this feeling before competition.

4) The Competition Cognitive Trait Anxiety Inventory (CCTAI) [16]. The questionnaire contains 33 questions, such as ‘Pay attention to the weather on the day of the competition’, with scores ranging from 1 to 4 points (from conforming to not conforming) determining by the closeness of the perception.

Pre-competition anxiety increases with the growing of scores on the aforementioned questionnaires.

### Collection of stool and urine samples

One week before the competition, the middle section of the first stool in the morning was collected in a 2 mL collection tube and frozen with liquid nitrogen. A total of 15 mL of the first urine in the morning was collected and centrifuged to obtain the supernatant. All of the above samples were stored in a -80 °C refrigerator before detection.

### Detection and characterization of gut microbiota

The Illumina Novaseq 6000 platform was used for the high-throughput sequencing of the 16 S rRNA V3-V4 region in stool samples (PE250 sequencing strategy). Bioinformatics methods were utilised to examine the differences in microbiota diversity and abundance. Sequences were clustered at 97% similarity, and 0.005% of all sequences were applied as the threshold filtering operational taxonomic units (OTUs). Alpha and beta diversity analyses and linear discriminant analysis (LDA) effect size (LEfSe) to identify species with significant differences were conducted. Redundancy analysis/canonical correspondence analysis (RDA/CCA) was applied to examine the relationship between different microbiota and anxiety levels.

### Analysis of gut and urine metabolites

A total of 50 mg of stool and 100 µL of urine samples were weighed and extracted with 1000 and 500 µL of 2-chlorophenylalanine in formaldehyde configuration, respectively. A liquid chromatography–mass spectrometry system was used for metabolomics analysis (Waters Acquity I-Class PLUS ultrahigh performance liquid chromatography in series with Waters Xevo G2-XS QTof high-resolution mass spectrometer). Progenesis QI software was applied to extract and align peaks from raw data collected by MassLynx V4.2. Data quality assessment and annotation, differential expression and functional enrichment analyses were performed on the basis of the test results.

### Statistical analysis

All the data were analyzed using SPSS20.0 and expressed as mean ± standard deviation. Student’s t-test was used to determine the statistical difference between two groups. The relationships between differential metabolites and pre-competition anxiety markers were analyzed through Spearman’s rank test. *P* < 0.05 was considered statistically significant.

## Results

### Levels of self-reported pre-competition anxiety

**Fig. 1:**
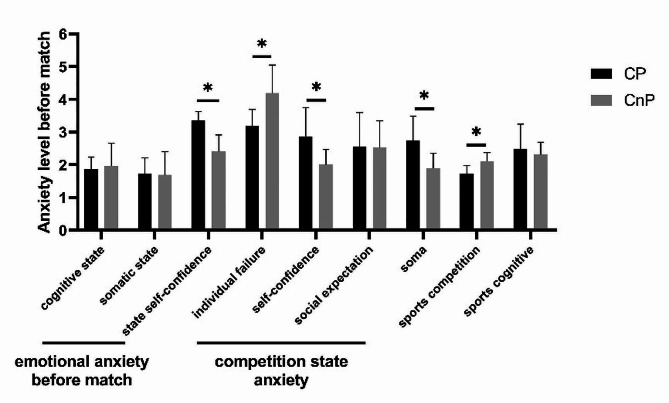
Wrestlers’ pre-competition anxiety levels (***n*** **= 6).** *Significant difference from CnP group

No significant difference in cognitive and physical state anxieties was found between the two groups. The competition state anxiety and state self-confidence of the CP group were significantly higher than those of the CnP group (*P* < 0.05). Individual failure anxiety in the CP group was lower than that in the CnP group (*P* < 0.05). Self-confidence and physical anxiety in the CP group were higher than those in the CnP group (*P* < 0.05). No significant difference in social expectation anxiety was found between the two groups. Competition anxiety in the CP group was lower than that in the CnP group (*P* < 0.05). No significant difference in cognitive trait anxiety was found between two groups (Fig. 1).

### Microbiome in faecal samples

**Fig. 2:**
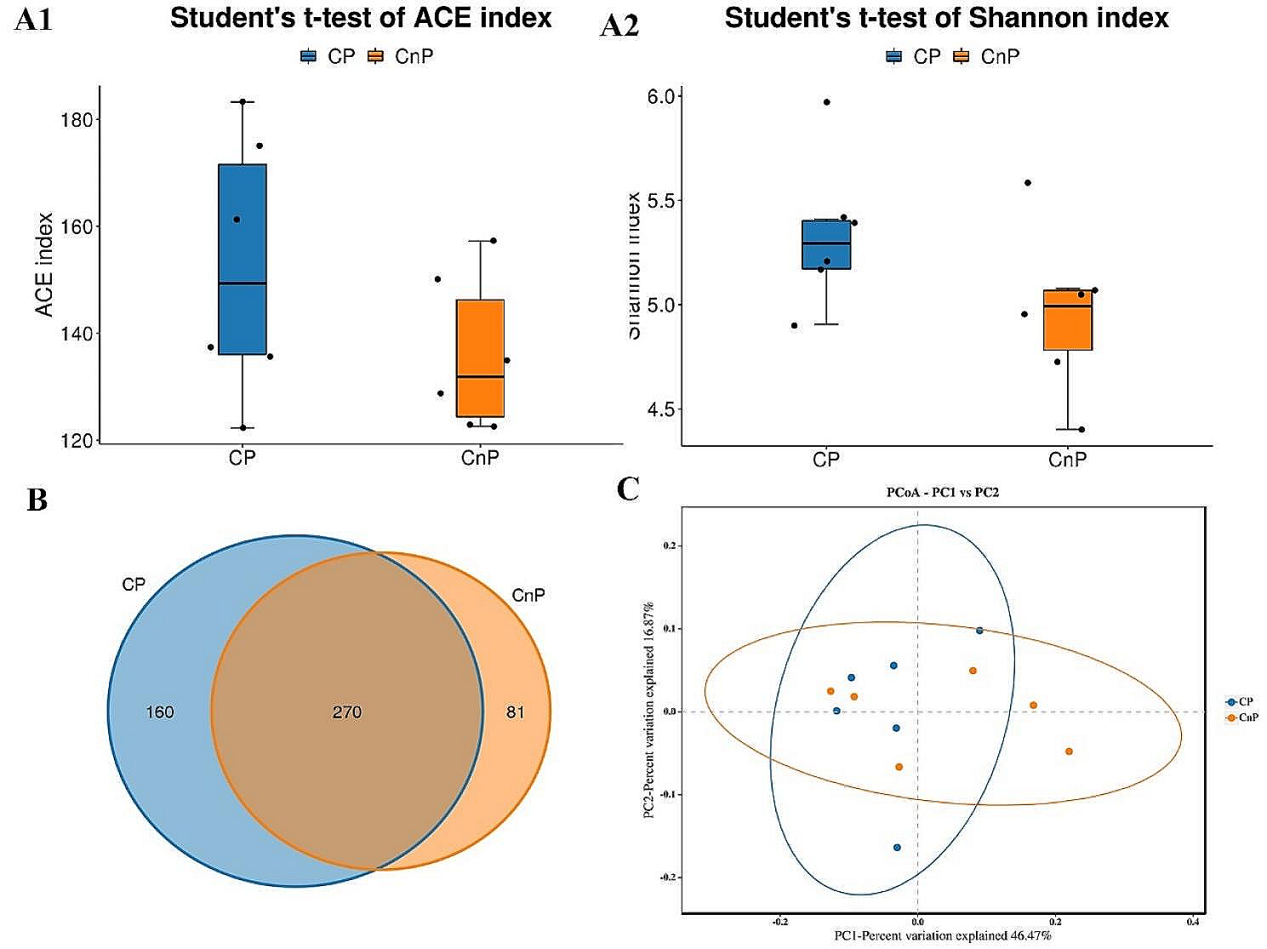
Diversity indices (α and β) of gut microbial species in the two groups (***n*** **= 6).** (**A1**) ACE index of Alpha diversity. (**A2**) shannon index of alpha diversity. (**B**) venn diagram of the α diversity distribution of OTUs. (**C**) PCoA graph of microbial beta diversity

Comparing the two groups’ α diversity indices revealed no discernible change in the ACE (Fig. 2A1) or Shannon (Fig. 2A2) indices. The CP group had higher indices than the CnP group. However, this difference was not discernible. A total of 511 OTUs were collected at a cut-off value of 3%. A total of 270 OTUs were found in both groups. Of these OTUs, 160 and 81 were found in the CP and CnP groups, respectively (Fig. 2B). The above data demonstrated that the gut microbiota in the CP group had a greater abundance and diversity than that in the CnP group. The gut microbiota diversity of the two groups varied, and PCoA showed that the CP group had a more concentrated β diversity distribution than the CnP group (Fig. 2C).

**Fig. 3:**
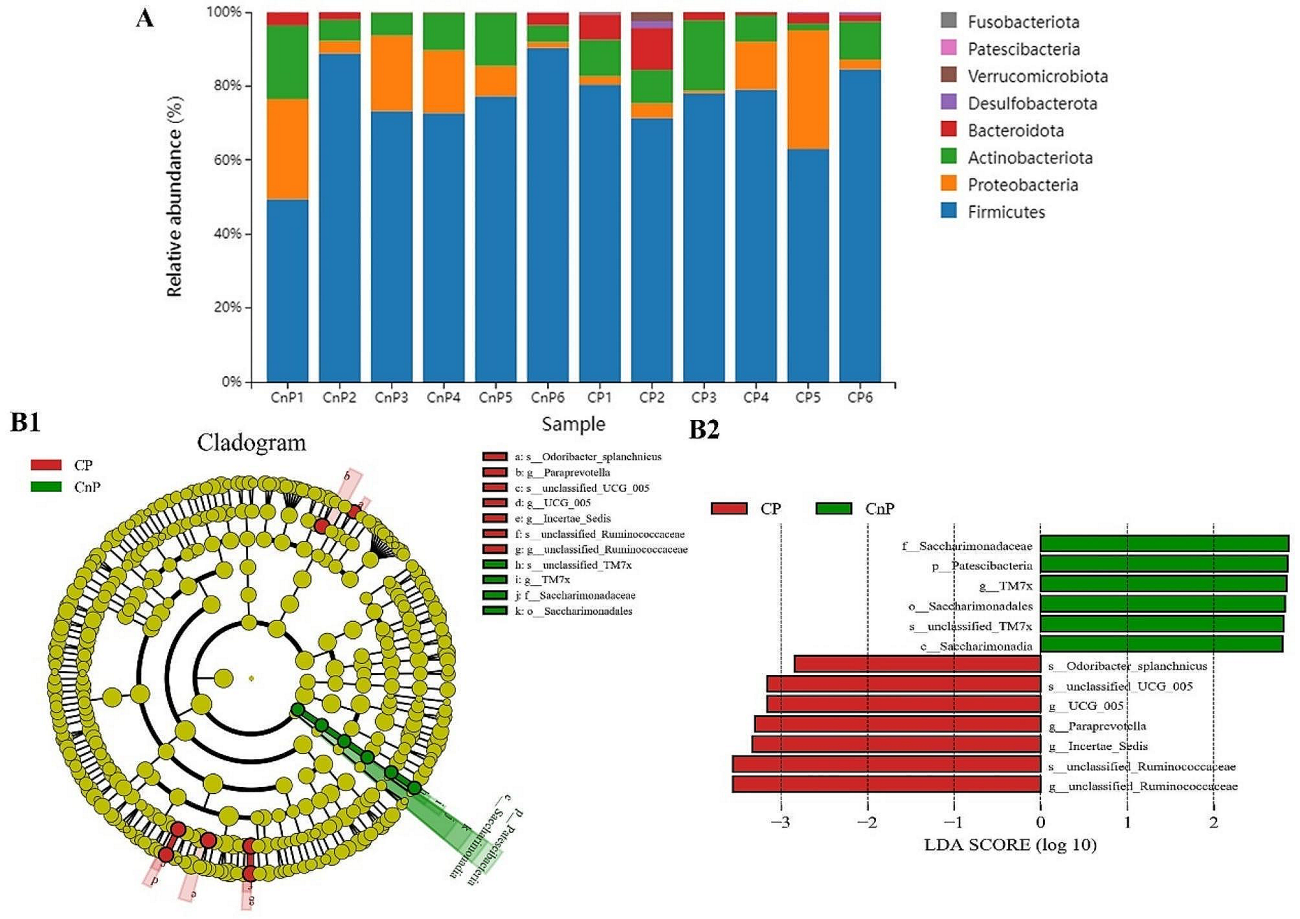
Gut microbial species distribution and significance analysis of differences between two groups (***n*** **= 6).** (**A**) Histogram of species distribution. (**B1**) evolutionary cladograms based on lefse analysis. (**B2**) histogram of the distribution of lda values. The letters p, c, o, f, g, and s stand for phylum, class, order, and species, respectively

Figure 3A displays the species distribution. The majority of the bacteria in the two groups belonged to the following phyla: *Fusobacteriota*, *Patescibacteria*, *Verrucomicrobia*, *Desulffobacterota*, *Bacteroidota*, *Actinobacteriota*, *Proteobacteria*, and *Firmicutes.* The taxonomic differences in the gut microbiota between the two groups were analysed by LEfSe. A total of 13 significantly different microbial groups (including 1 phylum, 1 class, 1 order, 1 family, 5 genera, and 4 species) were discovered in the two groups by using LDA ≥ 2.5 as the standard (Fig. 3B1). At the species level, *Oscillospiraceae UCG_005*, *Paraprevotella*, *Incertae Sedis*, and *Ruminococcaceae* were the differential bacteria in the CP group, and *Saccharobacter_TM7x* was the differential bacteria in the CnP group (Fig. 3B2).

### Metabolomics Analysis in Faecal and urine samples

**Fig. 4:**
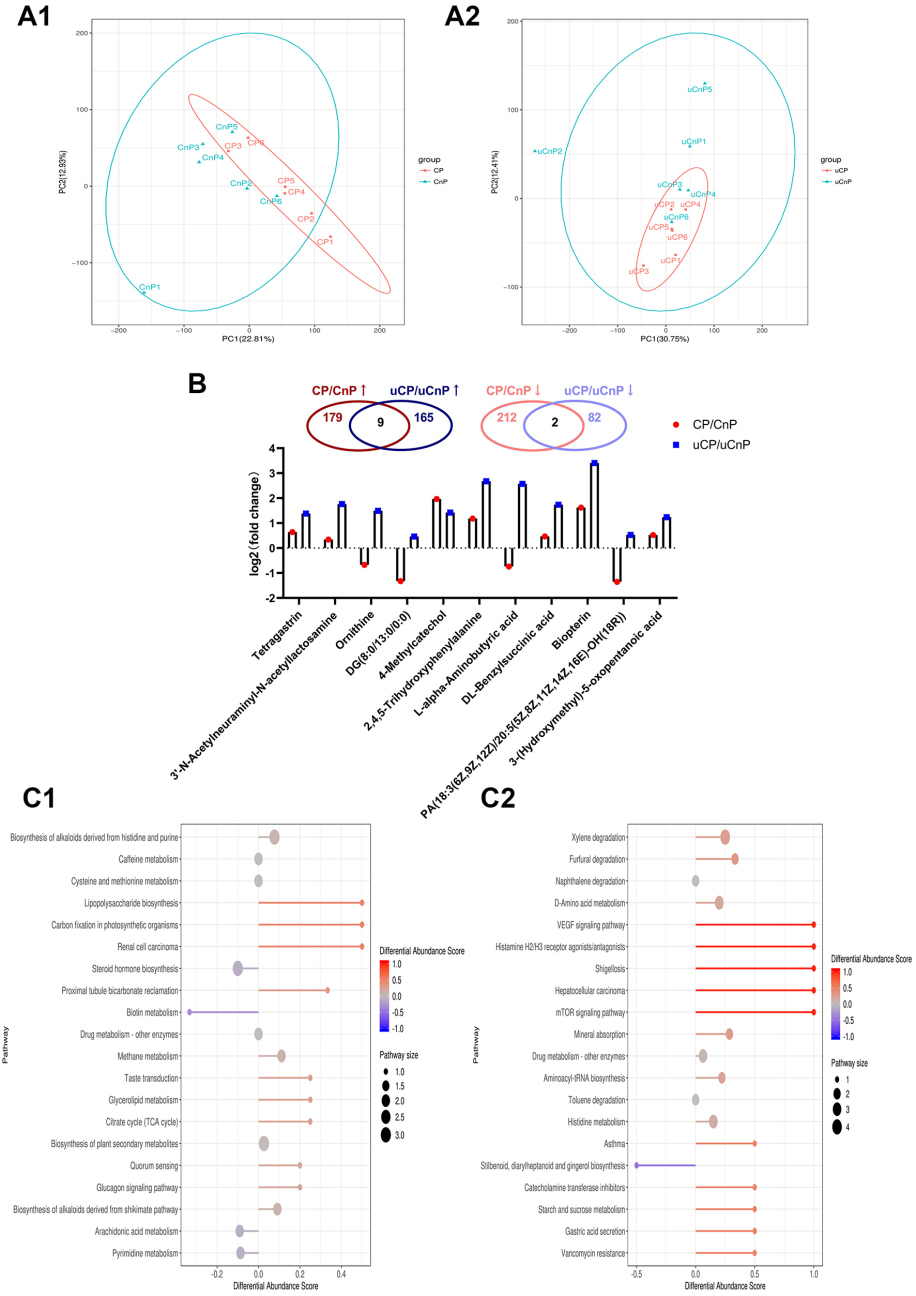
Comparative Metabolic Analysis of the Faecal and Urine Samples of the Two Groups (***n*** **= 6).** The two sets of faecal metabolites are indicated by CP/CnP, whereas those of urine metabolites are indicated as uCP/uCnP. PCA graph of metabolites in faecal (**A1**) and urine (**A2**) samples. (**B**) Venn diagram and histogram showing the fold-change in differential metabolites in faecal and urine samples. Differential metabolites that are up-regulated are indicated as CP/CnP↑ and uCP/uCnP↑, whereas those that are down-regulated are indicated as CP/CnP↓ and uCP/uCnP↓. Functional differential abundance score map showing the differential metabolites in faecal (**C1**) and urine (**C2**) samples. The differential pathway name is displayed on the ordinate, whereas the differential abundance score is displayed on the abscissa. The score is expressed as an absolute value based on the length of the line segment; scores close to 1 or − 1 indicate metabolites that are up- or down-regulated. The number of differential metabolites in the pathway is represented by the size of the dot; a dot close to red or blue indicates a low or high *P-value*

PCA revealed that the metabolite compositions in the faecal (Fig. 4A1) and urine (Fig. 4A2) samples of the two groups differed. A total of 402 differential metabolites were found in the faecal samples of the CP/CnP groups, comprising 188 up-regulated and 214 down-regulated metabolites. A total of 258 differential metabolites comprising 174 up-regulated and 84 down-regulated metabolites were found in the urine samples of the two groups. Nine of the 11 common differential metabolites found in faecal and urine samples were up-regulated (Fig. 4B). KEGG annotation revealed that the functions of the differential metabolites in faecal samples were primarily enriched in caffeine metabolism, histidine- and purine-derived alkaloid biosynthesis, LPS biosynthesis, biotin metabolism, glucagon signaling pathway and other processes (Fig. 4C1). Amino acid metabolism, histamine H2/H3 receptor agonists/antagonists, VEGF and the mTOR signaling pathway, mineral absorption, histidine metabolism, catecholamine transferase inhibitors, starch and sucrose metabolism, gastric acid secretion and other processes were amongst the functions of the primarily enriched differential microbial metabolites in urine samples (Fig. 4C2).

### Analysis of the correlation of Differential microorganisms and metabolites with anxiety indicators

**Fig. 5:**
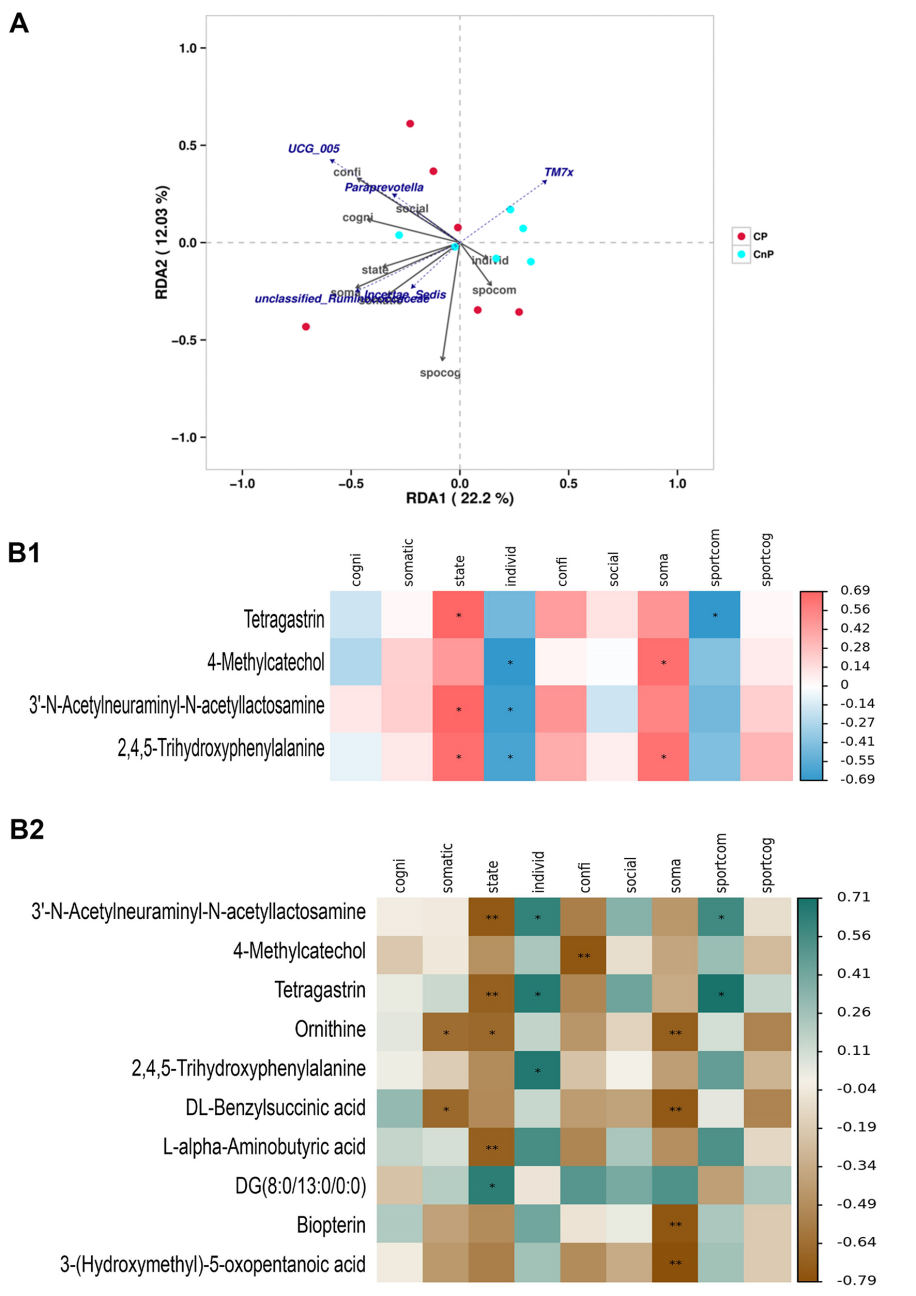
Correlation Analysis of Differential Microorganisms and Metabolites with Pre-Competition Anxiety Indicators (***n*** **= 6).** (**A**) The relationship between differential microorganisms and anxiety indicators was identified through RDA/CCA. The blue dotted line represents differential microorganisms, and the black solid line represents anxiety indices. The terms ‘cogni’, ‘somatic’, ‘state’, ‘individ’, ‘confi’, ‘social’, ‘soma’, ‘sportcom’ and ‘sportcog’ refer to anxiety related to the cognitive state, somatic state, state self-confidence, individual failure, self-confidence, social expectation, soma and sport traits, respectively. A long black arrow is indicative of the great effect of each anxiety index on differential microorganisms. A small angle between two arrows indicates a high correlation between anxiety indices and differential microorganisms. A sample close to the black arrow is indicative of the strong effect of an anxiety indicator on a sample. Samples in the same direction as the arrow indicate that an anxiety index is positively correlated with differential microoranisms and vice versa. Heat map of the correlation of differential metabolites in faecal (**B1**) and urine (**B2**) with anxiety levels. The differential metabolite is the ordinate and the anxiety index is the abscissa. Positive correlations are indicated by red/brown and negative correlations are indicated by blue/lake blue. *P < 0.05; **P < 0.01

*Oscillospiraceae (UCG_005)* and *Paraprevotella* were positively correlated with self-confidence and social expectation anxiety and negatively correlated with individual failure anxiety; somatic anxiety was positively associated with *Ruminococcus*_*unclassified* and inversely associated with *Saccharobacter_TM7x* (*P* < 0.05) (Fig. 5A). The analysis of the correlation between common differential metabolites in faecal samples and anxiety indicators revealed that tetrapeptide gastrin, 3ʹ-N-acetylneuraminyl-N-acetyllactosamine and 2,4,5-trihydroxyphenylalanine were positively correlated with state self-confidence; 4-methylcatechol, 3ʹ-*N*-acetylneuraminyl-*N*-acetyllactosamine and 2,4,5-trihydroxyphenylalanine were inversely associated with individual failure anxiety; 4-methylcatechol and 2,4,5-trihydroxyphenylalanine were positively associated with somatic anxiety; and tetragastrin was negatively associated with athletic competition anxiety (*P* < 0.05) (Fig. 5B1). Ornithine and DL-benzylsuccinic acid were positively correlated with body state anxiety. State self-confidence was positively correlated with 3ʹ-*N*-acetylneuraminyl-*N*-acetyllactosamine, tetrapeptide gastrin, ornithine and L-α-aminobutyric acid, and negative correlations were found between state self-confidence and DG (8:0/13:0/0:0). Amongst the metabolites in urine samples, 3ʹ-*N*-acetylneuraminyl-*N*-acetyllactosamine and tetragastrin were negatively correlated with individual failure and sports competition anxiety; 2,4,5-trihydroxyphenylalanine was negatively correlated with individual failure anxiety; 4-methylcatechol was positively correlated with self-confidence; and ornithine, DL-benzylsuccinate, biopterin and 3-(hydroxymethyl)-5-oxopentanoic acid were positively associated with somatic anxiety (*P* < 0.05) (Fig. 5B2).

## Discussion

The introduction of new regulations for wrestling competitions has presented challenges to the technical, tactical and mental abilities of wrestlers. Sustaining a suitable degree of nervousness prior to a wrestling match is beneficial for honing techniques and strategies and considerably influences the result of the matches. Pre-competition anxiety is more complicated than general anxiety, which can be measured directly with the Self-rated Anxiety Scale (SAS). Sports anxiety patterns can be dismantled into three main components: cognitive anxiety, somatic anxiety, and self-confidence [17]. The relationship between various anxiety indicators and competition performance differs.

Our study found that compared with the CnP group, the CP group exhibited higher levels of somatic anxiety and confidence and lower levels of anxiety related to athletic competition and individual failure. Somatic anxiety refers to the physiological and affective elements of the anxiety experience that develop directly from autonomic arousal. Self-confidence encompasses the general perception of achievement by competitors [17]. They have complex relationships with sport performance. While one study found a negative correlation between somatic anxiety and sports performance [18], another find a nonlinear relationship, wherein a certain amount of somatic anxiety may even be beneficial to sports performance [2]. Combat sports require a certain level of self-confidence, indicating that athletes enter competitions in a certain state and that self-confidence is essential for achieving good sports performance [19]. Research has demonstrated that sports self-confidence has a mediating effect on sports state anxiety and perceived performance, that is, sports self-confidence can help athletes manage their anxiety well and help them achieve their goals [20]. Cognitive anxiety is defined as the mental component of anxiety and is caused by negative expectations about success or by negative self-evaluation. Individual failure anxiety belongs to the category of cognitive anxiety, which describes athletes’ propensity to worry that they will not be able to meet their own expectations or perform at their own level. Cognitive state anxiety and sports performance are typically negatively correlated [21]. A study found a positive relationship between plasma cortisol levels and cognitive anxiety as well as sports competition anxiety in judo athletes [22]. Cortisol is an important procatabolic hormone. In athletes, increases in cortisol levels decrease fitness and increase propensity to fatigue from exercise; these effects, in turn, negatively affect motivation and competitiveness [23].

Although numerous psychological questionnaires and scales have long been applied to reflect athletes’ pre-competition anxiety levels, information linking the perception of anxiety and physiological/metabolic manifestations under stressful conditions in sports remains scarce [21, 24]. Exercise-induced stress responses may be controlled by gut microbiota, and a strong correlation exists between the composition of gut microbiota and physical and psychological stress experienced during exercise [25]. Psychological stress could result in gastrointestinal dysfunction and increased intestinal permeability, which can affect the composition and abundance of microbial communities. An athlete’s mood, motivation and fatigue level can be influenced by their gut microbiota, which also functions as an endocrine organ by stimulating the production of different neurotransmitters and hormones and regulating the HPA axis [26].

Our study found that *Oscillospiraceae (UCG_005)*, *Paraprevotella* and *Ruminococcaceae_unclassified* were more abundant in the CP group than in the CnP group, whereas *Saccharobacterium_TM7x* was more abundant in the CnP group than in the CP group. *Oscillospiraceae (UCG_005)* and *Paraprevotella* were positively correlated with self-confidence and social expectation anxiety and negatively correlated with individual failure anxiety. Research has demonstrated a strong positive correlation between *Oscillospiraceae* and social skills, including extroversion, communication abilities and comprehensive measurement [27]. Depressed patients and mice with decreased *Paraprevotella* abundance present increased susceptibility to stress and reduced sociability [28]. Our study also found that somatic anxiety was positively correlated with *Ruminococcaceae* and negatively correlated with *TM7x.* Depressed mice have been reported to exhibit higher *Ruminococcus* abundance and are more likely to experience anxiety reactions in response to long-term, unpredictable mild stress than control mice [29]. Depressed patients have low *Saccharibacteria_TM7x* abundance and exhibit higher levels of anxiety [30]. Probiotic supplements, such as *Lactobacilli* and *Bifidobacteria*, have been demonstrated to help athletes and the general public feel less stressed and anxious [7, 31, 32], However, whether this effect is connected to the increase in microorganisms in our study is unclear. This result could be connected to the variations in the gut microbiota in athletes participating in various sports. We present the first-ever report on pre-competition anxiety and gut microbiota in wrestlers. Low abundances of *Oscillospiraceae (UCG_005)* and *Paraprevotella* were found to be associated with negative emotions. This association could account for the higher precompetition anxiety and poorer sports performance of the CnP group than those of the CP group.

The brain-gut axis is the result of two-way communication between the enteric and autonomic nervous systems in the gastrointestinal tract. In addition to neuronal connections and gut microbiota, gastrointestinal hormones play a key role in brain–gut axis communication [33]. Moreover, in wrestlers undergoing pre-competition preparation and weight control, acute physical exertion exceeding 60% of maximum oxygen uptake (VO_2_max) may cause the HPA axis to release catabolic hormones. Exercise-induced psychological stress is linked to elevated levels of stress hormones in wrestlers [34]. Thus, we identified the kinds and concentrations of metabolites in the wrestlers’ faeces and urine prior to competition.

In our study, 11 common metabolites in faeces and urine were found, the majority of which were up-regulated (in other words, their contents were higher in the CP group than in the CnP group). Ornithine and L-α-aminobutyric acid are two metabolites with antianxiety properties and positively correlated with self-confidence [35]. In addition, ornithine may help improve lipid metabolism and reduce exercise fatigue [36].

We also analysed the functionality of the enriched faecal and urine metabolites. The functions of the up-regulated differential metabolites in the faeces of the CP group were enriched in histamine receptor processes and lipopolysaccharide (LPS) biosynthesis. The overproduction of stress hormones induced by physical and psychological stresses can cause LPS to translocate outside the gastrointestinal tract, trigger immune and inflammatory responses and lead to increased levels of histamine and proinflammatory cytokines in the circulation system. This phenomenon is known as leaky gut syndrome [37]. We further examined the metabolites in the urine of the CP group and did not find inflammatory factors. This result could be related to the enrichment of up-regulated metabolites in the VEGF pathway, which inhibits the increase in intestinal permeability, relieves exercise-induced ischaemia and encourages angiogenesis [38]. Therefore, while the wrestlers in the CP group went through a period of physical and psychological stress in the lead-up to competition, their level of anxiety was manageable and did not result in considerable alterations in physiological indicators. Moreover, the urine metabolites in the CP group were enriched in caffeine metabolism. In addition to promoting fat breakdown, caffeine can relieve fatigue, stimulate adrenaline release, save glycogen and excite the central nervous system [39].

We further analyzed the enriched pathways of differential metabolites and found that compared with those in the CnP group, the up-regulated metabolites in the CP group were enriched in the tricarboxylic acid (TCA) cycle and mTOR pathway. The mTOR pathway is crucial for protein synthesis and plays a major role in gaining more skeletal muscle mass [40]. A muscle-gut axis theory has been proposed on the basis of the numerous studies showing a strong correlation between gut microbiota and muscle mass. This theory suggests that the diversity and composition of gut microbiota may influence the metabolism and function of skeletal muscle [41]. Moderate exercise may enhance muscle glucose uptake, glycogen synthesis and protein synthesis. Its mechanism of action may be associated with the function of microbiota metabolites, such as short-chain fatty acids (SCFAs), which support the TCA cycle, accelerate energy delivery, improve muscle blood flow and enhance muscle function [42].

Naturally, this study has certain limitations. We were unable to identify cause-and-effect relationships amongst gut microbiota, pre-competition and sports performance because this study is cross-sectional. Additionally, all of the chosen subjects were elite athletes and had to be members of the same team and compete in the same event. Although we recruited all of the eligible subjects for this study, its sample size is still small. We will increase the number of samples in subsequent studies.

## Conclusions

High levels of self-confidence and appropriate somatic state anxiety before competition were found in wrestlers who performed well. Anxiety about sports competition and individual failure were greater in wrestlers who performed poorly than in those who performed well. Wrestlers with varying sports performances had different gut microbiota distributions and metabolite levels. Pre-competition anxiety was closely associated with gut microbiota and metabolite levels.

## Data Availability

The raw sequence data in the study has been deposited in the NCBI database and can be accessed via web links (https://www.ncbi.nlm.nih.gov/bioproject/PRJNA1083021).
